# Dynamics of Hopfield-Type Neural Networks with Modulo Periodic Unpredictable Synaptic Connections, Rates and Inputs

**DOI:** 10.3390/e24111555

**Published:** 2022-10-29

**Authors:** Marat Akhmet, Madina Tleubergenova, Akylbek Zhamanshin

**Affiliations:** 1 Department of Mathematics, Middle East Technical University, Ankara 06531, Turkey; 2 Department of Mathematics, Aktobe Regional University, Aktobe 030000, Kazakhstan; 3 Institute of Information and Computational Technologies CS MES RK, Almaty 050010, Kazakhstan

**Keywords:** Hopfield-type neural networks, modulo periodic unpredictable synaptic connections, rates and inputs, unpredictable solutions, exponential stability, numerical simulations

## Abstract

In this paper, we rigorously prove that unpredictable oscillations take place in the dynamics of Hopfield-type neural networks (HNNs) when synaptic connections, rates and external inputs are modulo periodic unpredictable. The synaptic connections, rates and inputs are synchronized to obtain the convergence of outputs on the compact subsets of the real axis. The existence, uniqueness, and exponential stability of such motions are discussed. The method of included intervals and the contraction mapping principle are applied to attain the theoretical results. In addition to the analysis, we have provided strong simulation arguments, considering that all the assumed conditions are satisfied. It is shown how a new parameter, degree of periodicity, affects the dynamics of the neural network.

## 1. Introduction

It is well-known that HNNs [1,2] are widely used in the fields of signal and image processing, pattern recognition, associative memory and optimization computation, among others [3,4,5,6,7,8]. Hence, they have been the object of intensive analysis by numerous authors in recent decades. With the increasing improvement in neural networks, the aforementioned systems are being modernized, and the dynamics of models with various types of coefficients are being investigated [9,10,11,12,13]. Special attention is being paid to the problem of the existence and stability of periodic and almost periodic solutions of HNNs [14,15,16,17,18,19,20,21], for which appropriate coefficients and conditions are necessary.

A few years ago, the boundaries of the classical theory of dynamical systems, founded by H. Poincare [22] and G. Birkhoff [23], were expanded by the concepts of unpredictable points and unpredictable functions [24]. It was proven that the unpredictable point leads to the existence of chaos in quasi-minimal sets. That is, the proof of the unpredictability simultaneously confirms Poincare chaos. Unpredictable functions were defined as unpredictable points in the Bebutov dynamical system [25], where the topology of convergence on compact sets of the real axis is used instead of the metric space. The use of such convergence significantly simplifies the problem of proving the existence of unpredictable solutions for differential equations and neural networks, and a new method of included intervals has been introduced and developed in several papers [26,27,28,29,30,31].

Let us commence with the main definitions.

**Definition** **1** ([25])**.** *A uniformly continuous and bounded function ψ:R→R is unpredictable if there exist positive numbers $\epsilon_{0},\delta$ and sequences $t_{n},s_{n}$, both of which diverge to infinity such that $|\psi \left(t+t_{n}\right)-\psi \left(t\right)|\to 0$ as n→∞ uniformly on compact subsets of R and $|\psi \left(t+t_{n}\right)-\psi \left(t\right)|>\epsilon_{0}$ for each $t\in [s_{n}-\delta ,s_{n}+\delta ]$ and n∈N.*

In Definition 1, the sequences $t_{n},$$s_{n},$n=1,2,… are said to be the *convergence* and *divergence* sequences of the function ψ(t), respectively. We call the uniform convergence on compact subsets of R *the convergence property*, and the existence of the sequence $s_{n}$ and positive numbers $\epsilon_{0},\delta$ is called *the separation property*. It is known [32] that an unpredictable function without separation property is said to be a *Poisson stable* function.

Let us introduce a new type of unpredictable functions, which are important objects for investigation in the paper.

**Definition** **2.** The sum ϕ(t)+ψ(t) is said to be a *modulo periodic unpredictable* function if ϕ(t) is a continuous periodic function and ψ(t) is an unpredictable function.

In this study, we focus on the Hopfield-type neural network with two-component coefficients and inputs:

(1) $$x_{i}^{\prime }\left(t\right)=-\left(a_{i}\left(t\right)+b_{i}\left(t\right)\right)x_{i}\left(t\right)+\sum_{j=1}^{p}\left(c_{ij}\left(t\right)+d_{ij}\left(t\right)\right)f_{j}\left(x_{j}\left(t\right)\right)+u_{i}\left(t\right)+v_{i}\left(t\right),\;i=1,2,\ldots ,p,$$

where $x_{i}\left(t\right)$ stands for the state vector of the *i*th unit at time *t*. The synaptic connections, rates and external inputs are modulo periodic unpredictable; they consist of two components such that $a_{i}\left(t\right),$$c_{ij}\left(t\right),$$u_{i}\left(t\right)$ are periodic and $b_{i}\left(t\right),$$d_{ij}\left(t\right),$$v_{i}\left(t\right)$ are unpredictable. $c_{ij}\left(t\right)$ and $d_{ij}\left(t\right)$ denote components of the synaptic connection weights of the *j*th unit with the *i*th unit at time *t*; the functions $f_{j}\left(x_{j}\left(t\right)\right)$ denote the measures of activation to its incoming potentials of the unit *j* at time t.

Consider the convergence sequence $t_{n}$ of the unpredictable function ψ(t). For fixed real number ω>0, one can write that $t_{n}\equiv \tau_{n}\left(mod\;\omega \right),$ where $0\leq \tau_{n}<\omega$ for all n≥1. The boundedness of the sequence $\tau_{n}$ implies that there exists a subsequence $\tau_{n_{l}},$ which converges to a number $\tau_{\omega },$ $0\leq \tau_{\omega }<\omega .$ That is, there exists a subsequence $t_{n_{l}}$ of the convergence sequence $t_{n}$ and a number $\tau_{\omega }$ such that $t_{n_{l}}\to \tau_{\omega }\left(mod\;\omega \right)$ as l→∞. We called the number $\tau_{\omega }$ the *Poisson shift* for the convergence sequence $t_{n}$ with respect to the ω [33]. Denote by $T_{\omega }$ the set of all Poisson shifts. The number $\kappa_{\omega }=inf\;T_{\omega },$ $0\leq \kappa_{\omega }<\omega ,$ is said to be *the Poisson number* for the convergence sequence $t_{n}.$ If $\kappa_{\omega }=0,$ then we say that the sequence $t_{n}$ satisfies the *kappa property.*

## 2. Methods

Due to the development of neural networks and their applications, classical types of functions such as periodic and almost periodic are no longer sufficient to study their dynamics. This is especially seen in analysis of the chaotic behavior of the systems. Therefore, in order to meet requirements of progress, many more functions are needed. To satisfy the demands, we have combined periodic and unpredictable components in rates and inputs. If the periodicity is inserted to serve for stability, the unpredictability guarantees chaotic dynamics. According to Definition 1, verification of the convergence and separation properties is necessary to prove the existence of unpredictable solutions. To provide constructive conditions for the existence of unpredictable solutions, we have determined the special *kappa property* of the convergence sequence $t_{n},$ with respect to the period ω.

The *method of included intervals*, which was introduced in paper [26] and has been developed in [27,28,29,33], is a powerful instrument for verifying convergence properties. This technique has been applied in the study of continuous unpredictable solutions of Hopfield-type neural networks with delayed and advanced arguments [30] and in the study of discontinuous unpredictable solutions of impulsive neural networks with Hopfield structures [31]. All the previous models in [30,31] are considered with constant rates, while in the present research, the rates are variable, and we have designed the new model of Hopfield-type neural networks with modulo periodic unpredictable rates $a_{i}\left(t\right)+b_{i}\left(t\right)$, connection weights $c_{ij}\left(t\right)+d_{ij}\left(t\right)$ and external inputs $u_{i}\left(t\right)+v_{i}\left(t\right).$ The periodic components, $a_{i}\left(t\right),$ serve the stability of the model, while the unpredictable components $b_{i}\left(t\right)$ and $v_{i}\left(t\right)$ cause chaotic outputs.

## 3. Main Results

Throughout the paper, we will use the norm $∥v∥=\max_{1\leq i\leq p}\left|v_{i}\right|,$ where $\left|\cdot \right|$ is the absolute value, $v=(v_{1},\ldots ,v_{p})$ and $v_{i}\in R,i=1,2,\ldots ,p$.

Following the results in [34], it can be shown that the function $y\left(t\right)=(y_{1}\left(t\right),y_{2}\left(t\right),\ldots ,y_{p}\left(t\right))$ is a solution of (1) if and only if it satisfies the following integral equation:

(2) $$\begin{array}{ccc} & & y_{i}\left(t\right)=-\int_{-\infty }^{t}e^{-\int_{s}^{t}a_{i}\left(u\right)du}\left(-b_{i}\left(s\right)y_{i}\left(s\right)+\sum_{j=1}^{p}\left(c_{ij}\left(s\right)+d_{ij}\left(s\right)\right)f_{j}\left(y_{j}\left(s\right)\right)+u_{i}\left(s\right)+v_{i}\left(s\right)\right)ds, \end{array}$$

for all i=1,…,p.

Denote by S the set of vector-functions $\varphi =(\varphi_{1},\varphi_{2},\ldots ,\varphi_{p}),$ where $\varphi_{i},$ i=1,2,…,p, satisfy the convergence property with the common convergence sequence $t_{n}.$ Moreover, $|\varphi_{i}|<H,$ i=1,2,⋯,p, where *H* is a positive number. In the set, S determines the norm ${∥\varphi \left(t\right)∥}_{0}=\max_{\left(i\right)}\left|\varphi_{i}\left(t\right)\right|.$

Define on S the operator *T* such that $T\phi \left(t\right)=(T_{1}\phi \left(t\right),T_{2}\phi \left(t\right),\ldots ,T_{p}\phi \left(t\right))$, where:

(3) $$\begin{array}{ccc} & & T_{i}\phi \left(t\right)\equiv -\int_{-\infty }^{t}e^{-\int_{s}^{t}a_{i}\left(u\right)du}\left(-b_{i}\left(s\right)\phi_{i}\left(s\right)+\sum_{j=1}^{p}\left(c_{ij}\left(s\right)+d_{ij}\left(s\right)\right)f_{j}\left(\phi_{j}\left(s\right)\right)+u_{i}\left(s\right)+v_{i}\left(s\right)\right)ds, \end{array}$$

for each i=1,2,…, p. We will need the following conditions:

(C1) The functions $a_{i}\left(t\right),$ $c_{ij}\left(t\right)$ and $u_{i}\left(t\right)$ are continuous ω—periodic, and $\int_{0}^{\omega }a_{i}\left(u\right)du>0$ for each i,j=1,…,p;
(C2) The functions $b_{i}\left(t\right),$ $d_{ij}\left(t\right)$ and $v_{i}\left(t\right),$ i,j=1,2,…,p, are unpredictable with the same convergence and divergence sequences $t_{n},\;s_{n}.$ Moreover, $|v_{i}\left(t+t_{n}\right)-v_{i}\left(t\right)|>\epsilon_{0}$ for all $t\in [s_{n}-\delta ;s_{n}+\delta ],$i=1,2,…,p, and positive numbers δ,$\epsilon_{0};$
(C3) The convergence sequence $t_{n}$ satisfies the kappa property with respect to the period ω;
(C4) There exists a positive number $m_{f}$ such that $\sup_{|s|<H}\left|f\left(s\right)\right|=m_{f};$
(C5) There exists a positive number *L* such that the function f(s) satisfies the inequality $|f\left(s_{1}\right)-f\left(s_{2}\right)\left|\;\leq \;L\right|s_{1}-s_{2}|$ if $|s_{1}\left|<H,\right|s_{2}|<H.$

According the condition (C1), for all i=1,…, p, the numbers $K_{i}\leq 1$ and $\lambda_{i}>0$ exist, such that

(4) $$\begin{array}{c} e^{-\int_{s}^{t}a_{i}\left(u\right)du}\leq K_{i}e^{-\lambda_{i}\left(t-s\right)}. \end{array}$$

For convenience, we introduce the following notations:

$$\begin{array}{ccc} & & m_{i}^{a}=\underset{t\in R}{\sup}|a_{i}\left(t\right)|,\;m_{i}^{b}=\underset{t\in R}{\sup}|b_{i}\left(t\right)|,\;m_{i}^{u}=\underset{t\in R}{\sup}\left|u_{i}\left(t\right)\right|,\\ & & m_{i}^{v}=\underset{t\in R}{\sup}|v_{i}\left(t\right)|,\;m_{ij}^{c}=\underset{t\in R}{\sup}|c_{ij}\left(t\right)|,\;m_{ij}^{d}=\underset{t\in R}{\sup}\left|d_{ij}\left(t\right)\right|, \end{array}$$

for each i=1,2,…,p.

The following conditions are required:

(C6) $\frac{K_{i}}{\lambda_{i}-K_{i}m_{i}^{b}}\left(\sum_{j=1}^{p}\left(m_{ij}^{c}+m_{ij}^{d}\right)m_{f}+m_{i}^{u}+m_{i}^{v}\right)<H$;
(C7) $K_{i}\left(m_{i}^{b}+\sum_{j=1}^{p}\left(m_{ij}^{c}+m_{ij}^{d}\right)L\right)<\lambda_{i}$;
(C8) $Hm_{i}^{b}+\sum_{j=1}^{p}m_{ij}^{d}m_{f}<\frac{\epsilon_{0}}{4}$;

for all i,j=1,…,p.

**Lemma** **1.** *The set S is a complete space.*

**Proof.**  Consider a Cauchy sequence $\phi^{k}\left(t\right)$ in S, which converges to a limit function ϕ(t) on R. Fix a closed and bounded interval I⊂R. We obtain:

(5) $$\begin{array}{c} ∥\phi \left(t+t_{n}\right)-\phi \left(t\right)∥\;\leq \;∥\phi \left(t+t_{n}\right)-\phi^{k}\left(t+t_{n}\right)∥\;+\;∥\phi^{k}\left(t+t_{n}\right)-\phi^{k}\left(t\right)∥\;+\;∥\phi^{k}\left(t\right)-\phi \left(t\right)∥. \end{array}$$

One can choose sufficiently large *n* and k, such that each term on the right side of (5) is smaller than $\frac{\epsilon }{3}$ for an arbitrary ϵ>0 and t∈I. Thus, we conclude that the sequence $\phi (t+t_{n})$ is uniformly converging to ϕ(t) on I. That is, the set S is complete. □

**Lemma** **2.** *The operator T is invariant in S.*

**Proof.**  For a function φ(t)∈S and fixed i=1,2,…,p, we have that

$$\begin{array}{ccc} |T_{i}\varphi \left(t\right)| & = & \left|\int_{-\infty }^{t}\;e^{-\int_{t}^{s}a_{i}\left(u\right)du}\left[-b_{i}\left(s\right)\varphi_{i}\left(s\right)+\sum_{j=1}^{p}\;\left(c_{ij}\left(s\right)+d_{ij}\left(s\right)\right)f_{j}\left(\varphi_{j}\left(s\right)\right)+u_{i}\left(s\right)+v_{i}\left(s\right)\right]ds\right|\\ & \leq & \int_{-\infty }^{t}\;K_{i}e^{-\lambda_{i}\left(t-s\right)}\left[|b_{i}\left(s\right)\varphi_{i}\left(s\right)|+\sum_{j=1}^{p}\;(|c_{ij}\left(s\right)|+|d_{ij}\left(s\right)|)|f_{j}\left(\varphi_{j}\left(s\right)\right)\left|+\right|u_{i}\left(s|+\right|v_{i}\left(s\right)|\right]ds\\ & \leq & \frac{K_{i}}{\lambda_{i}}\left(m_{i}^{b}H+\sum_{j=1}^{p}\left(m_{ij}^{c}+m_{ij}^{d}\right)m_{f}+m_{i}^{u}+m_{i}^{v}\right). \end{array}$$

The last inequality and condition (C6) imply that ${∥T\varphi ∥}_{0}<H.$

Next, applying the method of included intervals, we will show that $T\varphi \left(t+t_{n}\right)\to T\varphi \left(t\right)$ as n→∞ uniformly on compact subsets of R.

Let us fix an arbitrary ϵ>0 and a section [α,β], −∞<α<β<∞. There exist numbers γ, ξ such that γ<α and ξ>0, which satisfy the following inequalities:

(6) $$\frac{K_{i}}{\lambda_{i}}e^{-\lambda_{i}\left(\alpha -\gamma \right)}\left(m_{i}^{b}H+\frac{1}{4}\sum_{j=1}^{p}\left(m_{ij}^{c}+m_{ij}^{d}\right)\left(LH+m_{f}\right)+m_{i}^{u}+m_{i}^{v}\right)<\frac{\epsilon }{8},$$

(7) $$\frac{K_{i}}{\lambda_{i}}\left(e^{\xi (\beta -\gamma )}-1\right)\left(m_{i}^{b}H+\sum_{j=1}^{p}\left(m_{ij}^{c}+m_{ij}^{d}\right)m_{f}+m_{i}^{u}+m_{i}^{v}\right)<\frac{\epsilon }{4},$$

and

(8) $$\frac{K_{i}\xi }{\lambda_{i}}\left(m_{i}^{b}+H+\sum_{j=1}^{p}\left(m_{ij}^{c}+m_{ij}^{d}\right)L+2pm_{f}+2\right)<\frac{\epsilon }{4},$$

for all i=1,2,…,p.

Since the functions $b_{i}\left(t\right),$ $d_{ij}\left(t\right)$ and $v_{i}\left(t\right),$ i,j=1,2,…,p, are unpredictable, φ(t) belongs to S, and the convergence sequence, $t_{n},$ is common to all of them and satisfies the kappa property. Then, the following inequalities are true: $|b_{i}\left(t+t_{n}\right)-b_{i}\left(t\right)|<\xi ,$ $|d_{ij}\left(t+t_{n}\right)-d_{ij}\left(t\right)|<\xi ,$ $|v_{i}\left(t+t_{n}\right)-v_{i}\left(t\right)|<\xi ,$ $|\varphi_{i}\left(t+t_{n}\right)-\varphi_{i}\left(t\right)|<\xi$ for t∈[γ,β]. Moreover, applying condition (C3), one can attain that $|a_{i}\left(t+t_{n}\right)-a_{i}\left(t\right)|<\xi ,$ $|c_{ij}\left(t+t_{n}\right)-c_{ij}\left(t\right)|<\xi ,$ and $|u_{i}\left(s+t_{n}\right)-u_{i}\left(s\right)|<\xi$ for t∈R, i,j=1,2,…,p. We have that:

$$\begin{array}{ccc} & & |T_{i}\varphi \left(t+t_{n}\right)-T_{i}\varphi \left(t\right)|\;\leq \;|\int_{-\infty }^{t}e^{-\int_{s}^{t}a_{i}\left(u+t_{n}\right)du}(-b_{i}\left(s+t_{n}\right)\varphi_{i}\left(s+t_{n}\right)+\\ & & \sum_{j=1}^{p}\left(c_{ij}\left(s+t_{n}\right)+d_{ij}\left(s+t_{n}\right)\right)f_{j}\left(\varphi_{j}\left(s+t_{n}\right)\right)+u_{i}\left(s+t_{n}\right)+v_{i}\left(s+t_{n}\right))ds-\\ & & \int_{-\infty }^{t}e^{-\int_{s}^{t}a_{i}\left(u\right)du}\left(-b_{i}\left(s\right)\varphi_{i}\left(s\right)+\sum_{j=1}^{p}\left(c_{ij}\left(s\right)+d_{ij}\left(s\right)\right)f_{j}\left(\varphi_{j}\left(s\right)\right)+u_{i}\left(s\right)+v_{i}\left(s\right)\right)ds|\leq \\ & & \int_{-\infty }^{t}\left|e^{-\int_{s}^{t}a_{i}\left(u+t_{n}\right)du}-e^{-\int_{s}^{t}a_{i}\left(u\right)du}\right||-b_{i}\left(s+t_{n}\right)\varphi_{ij}\left(s+t_{n}\right)+\\ & & \sum_{j=1}^{p}\left(c_{ij}\left(s+t_{n}\right)+d_{ij}\left(s+t_{n}\right)\right)f_{j}\left(\varphi_{j}\left(s+t_{n}\right)\right)+u_{i}\left(s+t_{n}\right)+v_{i}\left(s+t_{n}\right)|ds+\\ & & \int_{-\infty }^{t}e^{-\int_{s}^{t}a_{i}\left(u\right)du}|-b_{i}\left(s+t_{p}\right)\varphi_{i}\left(s+t_{p}\right)+b_{i}\left(s\right)\varphi_{i}\left(s\right)+\\ & & \sum_{j=1}^{p}\;\left(c_{ij}\left(s+t_{n}\right)+d_{ij}\left(s+t_{n}\right)\right)\left(f_{j}\left(\varphi_{j}\left(s+t_{n}\right)\right)-f_{j}\left(\varphi_{j}\left(s\right)\right)\right)+\\ & & \sum_{j=1}^{p}\;\left(c_{ij}\left(s+t_{n}\right)-c_{ij}\left(s\right)+d_{ij}\left(s+t_{n}\right)-d_{ij}\left(s\right)\right)f_{j}\left(\varphi_{j}\left(s\right)\right)+u_{i}\left(s+t_{n}\right)+v_{i}\left(s+t_{n}\right)-u_{i}\left(s\right)-v_{i}\left(s\right)|ds, \end{array}$$

for all i=1,2,…,p. Consider the terms in the last inequality separately on intervals (−∞,γ] and (γ,t]. By using inequalities (6)–(8), we obtain:

$$\begin{array}{ccc} & & I_{1}=\int_{-\infty }^{\gamma }\left|e^{-\int_{s}^{t}a_{i}\left(u+t_{n}\right)du}-e^{-\int_{s}^{t}a_{i}\left(u\right)du}\right||-b_{i}\left(s+t_{n}\right)\varphi_{ij}\left(s+t_{n}\right)+\\ & & \sum_{j=1}^{p}\;\left(c_{ij}\left(s+t_{n}\right)+d_{ij}\left(s+t_{n}\right)\right)f_{j}\left(\varphi_{j}\left(s+t_{n}\right)\right)+u_{i}\left(s+t_{n}\right)+v_{i}\left(s+t_{n}\right)|ds+\\ & & \int_{-\infty }^{\gamma }e^{-\int_{s}^{t}a_{i}\left(u\right)du}|-b_{i}\left(s+t_{p}\right)\varphi_{i}\left(s+t_{p}\right)+b_{i}\left(s\right)\varphi_{i}\left(s\right)+\\ & & \sum_{j=1}^{p}\;\left(c_{ij}\left(s+t_{n}\right)+d_{ij}\left(s+t_{n}\right)\right)\left(f_{j}\left(\varphi_{j}\left(s+t_{n}\right)\right)-f_{j}\left(\varphi_{j}\left(s\right)\right)\right)+\\ & & \sum_{j=1}^{p}\;\left(c_{ij}\left(s+t_{n}\right)-c_{ij}\left(s\right)+d_{ij}\left(s+t_{n}\right)-d_{ij}\left(s\right)\right)f_{j}\left(\varphi_{j}\left(s\right)\right)+u_{i}\left(s+t_{n}\right)+v_{i}\left(s+t_{n}\right)-u_{i}\left(s\right)-v_{i}\left(s\right)|ds\leq \\ & & \int_{-\infty }^{\gamma }2K_{i}e^{-\lambda_{i}\left(t-s\right)}\left(m_{i}^{b}H+\sum_{j=1}^{p}\left(m_{ij}^{c}+m_{ij}^{d}\right)m_{f}+m_{i}^{u}+m_{i}^{v}\right)ds+\\ & & \int_{-\infty }^{\gamma }K_{i}e^{-\lambda_{i}\left(t-s\right)}\left(2m_{i}^{b}H+\sum_{j=1}^{p}\left(m_{ij}^{c}+m_{ij}^{d}\right)LH+2\sum_{j=1}^{p}\left(m_{ij}^{c}+m_{ij}^{d}\right)m_{f}+2m_{i}^{u}+2m_{i}^{v}\right)ds\leq \\ & & \frac{2K_{i}}{\lambda_{i}}e^{-\lambda_{i}\left(\alpha -\gamma \right)}\left(m_{i}^{b}H+\sum_{j=1}^{p}\left(m_{ij}^{c}+m_{ij}^{d}\right)m_{f}+m_{i}^{u}+m_{i}^{v}\right)+\\ & & \frac{K_{i}}{\lambda_{i}}e^{-\lambda_{i}\left(\alpha -\gamma \right)}\left(2m_{i}^{b}H+\sum_{j=1}^{p}\left(m_{ij}^{c}+m_{ij}^{d}\right)\left(LH+2m_{f}\right)+2m_{i}^{u}+2m_{i}^{v}\right)\leq \\ & & \frac{4K_{i}}{\lambda_{i}}e^{-\lambda_{i}\left(\alpha -\gamma \right)}\left(m_{i}^{b}H+\frac{1}{4}\sum_{j=1}^{p}\left(m_{ij}^{c}+m_{ij}^{d}\right)\left(LH+m_{f}\right)+m_{i}^{u}+m_{i}^{v}\right)<\frac{\epsilon }{2}, \end{array}$$

and

$$\begin{array}{ccc} & & I_{2}=\int_{\gamma }^{t}\left|e^{-\int_{s}^{t}a_{i}\left(u+t_{n}\right)du}-e^{-\int_{s}^{t}a_{i}\left(u\right)du}\right||-b_{i}\left(s+t_{n}\right)\varphi_{ij}\left(s+t_{n}\right)+\\ & & \sum_{j=1}^{p}\;\left(c_{ij}\left(s+t_{n}\right)+d_{ij}\left(s+t_{n}\right)\right)f_{j}\left(\varphi_{j}\left(s+t_{n}\right)\right)+u_{i}\left(s+t_{n}\right)+v_{i}\left(s+t_{n}\right)|ds-\\ & & \int_{\gamma }^{t}e^{-\int_{s}^{t}a_{i}\left(u\right)du}|-b_{i}\left(s+t_{p}\right)\varphi_{i}\left(s+t_{p}\right)+b_{i}\left(s\right)\varphi_{i}\left(s\right)+\\ & & \sum_{j=1}^{p}\;\left(c_{ij}\left(s+t_{n}\right)+d_{ij}\left(s+t_{n}\right)\right)\left(f_{j}\left(\varphi_{j}\left(s+t_{n}\right)\right)-f_{j}\left(\varphi_{j}\left(s\right)\right)\right)+\\ & & \sum_{j=1}^{p}\;\left(c_{ij}\left(s+t_{n}\right)-c_{ij}\left(s\right)+d_{ij}\left(s+t_{n}\right)-d_{ij}\left(s\right)\right)f_{j}\left(\varphi_{j}\left(s\right)\right)+u_{i}\left(s+t_{n}\right)+v_{i}\left(s+t_{n}\right)-u_{i}\left(s\right)-v_{i}\left(s\right)|ds\\ & & \leq \int_{\gamma }^{t}K_{i}e^{-\lambda_{i}\left(t-s\right)}\left(e^{\xi (\beta -\gamma )}-1\right)\left(m_{i}^{b}H+\sum_{j=1}^{p}\left(m_{ij}^{c}+m_{ij}^{d}\right)m_{f}+m_{i}^{u}+m_{i}^{v}\right)ds+\\ & & \int_{\gamma }^{t}K_{i}e^{-\lambda_{i}\left(t-s\right)}\left(\left(m_{i}^{b}+H\right)\xi +\sum_{j=1}^{p}\left(m_{ij}^{c}+m_{ij}^{d}\right)L\xi +2\xi pm_{f}+2\xi \right)ds\leq \\ & & \frac{K_{i}}{\lambda_{i}}\left(e^{\xi (\beta -\gamma )}-1\right)\left(m_{i}^{b}H+\sum_{j=1}^{p}\left(m_{ij}^{c}+m_{ij}^{d}\right)m_{f}+m_{i}^{u}+m_{i}^{v}\right)+\\ & & \frac{K_{i}}{\lambda_{i}}\left(\left(m_{i}^{b}+H\right)\xi +\sum_{j=1}^{p}\left(m_{ij}^{c}+m_{ij}^{d}\right)L\xi +2\xi pm_{f}+2\xi \right)<\frac{\epsilon }{4}+\frac{\epsilon }{4}=\frac{\epsilon }{2}, \end{array}$$

for each i=1,2,…,p. This is why, for all t∈[α,β] and i=1,2,…,p, we have that $|T_{i}\varphi \left(t+t_{n}\right)-T_{i}\varphi \left(t\right)|\leq I_{1}+I_{2}<\epsilon .$ So, the function $T\varphi (t+t_{n})$ uniformly convergences to Tφ(t) on compact subsets of R, and it is true that T:S→S. □

**Lemma** **3.** *The operator T is contractive in S, provided that the conditions (C1)–(C7) are valid.*

**Proof.**  For two functions φ,ψ∈S, and fixed i=1,2,⋯,p, it is true that

$$\begin{array}{ccc} & & |T_{i}\varphi \left(t\right)-T_{i}\psi \left(t\right)|\leq \int_{-\infty }^{t}e^{-\int_{s}^{t}a_{i}\left(u\right)du}\left(\right|b_{i}\left(s\right)||\varphi_{i}\left(s\right)-\psi_{i}\left(s\right)|+\sum_{j=1}^{p}c_{ij}\left(s\right)\left|f_{j}\left(\varphi_{j}\left(s\right)\right)-f_{j}\left(\psi_{j}\left(s\right)\right)\right|ds+\\ & & \sum_{j=1}^{p}d_{ij}\left(s\right)|f_{j}\left(\varphi_{j}\left(s\right)\right)-f_{j}\left(\psi_{j}\left(s\right)\right)|ds)ds\leq \frac{K_{i}}{\lambda_{i}}(m_{i}^{b}|\varphi_{i}\left(s\right)-\psi_{i}\left(s\right)|+\sum_{j=1}^{p}m_{ij}^{c}L\left|\varphi_{j}\left(s\right)-\psi_{j}\left(s\right)\right|+\\ & & \sum_{j=1}^{p}m_{ij}^{d}L|\varphi_{j}\left(s\right)-\psi_{j}\left(s\right)|)ds\leq \frac{K_{i}}{\lambda_{i}}\left(m_{i}^{b}+\sum_{j=1}^{p}\left(m_{ij}^{c}+m_{ij}^{d}\right)L\right){∥\varphi -\psi ∥}_{0}. \end{array}$$

The last inequality yields ${∥T\varphi \left(t\right)-T\psi \left(t\right)∥}_{0}\leq \max_{i}\left(\frac{K_{i}}{\lambda_{i}}\left(m_{i}^{b}+\sum_{j=1}^{p}\left(m_{ij}^{c}+m_{ij}^{d}\right)L\right)\right){∥\varphi \left(t\right)-\psi \left(t\right)∥}_{0}$. Hence, in accordance with condition (C7), the operator *T* is contractive in S. □

**Theorem** **1.** *The neural network (1) admits a unique exponentially stable unpredictable solution provided that conditions (C1)–(C8) are fulfilled.*

**Proof.**  By Lemma 1, the set S is complete; by Lemma 2, the the operator *T* is invariant in S; and by Lemma 3, the operator *T* is contractive in S. Applying the contraction mapping theorem, we obtain that there exists a fixed point ω∈S of the operator T, which is a solution of the neural network (1) and satisfies the convergence property.

Next, we show that the solution ω(t) of (1) satisfies the separation property.

Applying the relations

$$\begin{array}{ccc} & & \omega_{i}\left(t\right)=\omega_{i}\left(s_{n}\right)-\int_{s_{n}}^{t}a_{i}\left(s\right)\omega_{i}\left(s\right)ds-\int_{s_{n}}^{t}b_{i}\left(s\right)\omega_{i}\left(s\right)ds+\int_{s_{n}}^{t}\sum_{j=1}^{p}c_{ij}\left(s\right)f_{j}\left(\omega_{j}\left(s\right)\right)ds+\\ & & \int_{s_{n}}^{t}\sum_{j=1}^{p}d_{ij}\left(s\right)f_{j}\left(\omega_{j}\left(s\right)\right)ds+\int_{s_{n}}^{t}u_{i}\left(s\right)ds+\int_{s_{n}}^{t}v_{i}\left(s\right)ds \end{array}$$

and

$$\begin{array}{ccc} & & \omega_{i}\left(t+t_{n}\right)=\omega_{i}\left(s_{n}+t_{n}\right)-\int_{s_{n}}^{t}a_{i}\left(s+t_{n}\right)\omega_{i}\left(s+t_{n}\right)ds-\int_{s_{n}}^{t}b_{i}\left(s+t_{n}\right)\omega_{i}\left(s+t_{n}\right)ds+\\ & & \int_{s_{n}}^{t}\sum_{j=1}^{p}c_{ij}\left(s+t_{n}\right)f_{j}\left(\omega_{j}\left(s+t_{n}\right)\right)ds+\int_{s_{n}}^{t}\sum_{j=1}^{p}d_{ij}\left(s+t_{n}\right)f_{j}\left(\omega_{j}\left(s\right)\right)ds+\int_{s_{n}}^{t}u_{i}\left(s+t_{n}\right)ds+\int_{s_{n}}^{t}v_{i}\left(s+t_{n}\right)ds \end{array}$$

we obtain:

$$\begin{array}{ccc} & & \omega_{i}\left(t+t_{n}\right)-\omega_{i}\left(t\right)=\omega_{i}\left(s_{n}+t_{n}\right)-\omega_{i}\left(s_{n}\right)-\int_{s_{n}}^{t}a_{i}\left(s+t_{n}\right)\left(\omega_{i}\left(s+t_{n}\right)-\omega_{i}\left(s\right)\right)ds-\\ & & \int_{s_{n}}^{t}\omega_{i}\left(s\right)\left(a_{i}\left(s+t_{n}\right)-a_{i}\left(s\right)\right)ds-\int_{s_{n}}^{t}b_{i}\left(s+t_{n}\right)\left(\omega_{i}\left(s+t_{n}\right)-\omega_{i}\left(s\right)\right)ds-\\ & & \int_{s_{n}}^{t}\omega_{i}\left(s\right)\left(b_{i}\left(s+t_{n}\right)-b_{i}\left(s\right)\right)ds+\int_{s_{n}}^{t}\sum_{j=1}^{p}c_{ij}\left(s+t_{n}\right)\left(f_{i}\left(\omega_{i}\left(s+t_{n}\right)\right)-f_{i}\left(\omega_{i}\left(s\right)\right)\right)ds+\\ & & \int_{s_{n}}^{t}\sum_{j=1}^{p}\left(c_{ij}\left(s+t_{n}\right)-c_{ij}\left(s\right)\right)f_{i}\left(\omega_{i}\left(s\right)\right)ds+\int_{s_{n}}^{t}\sum_{j=1}^{p}d_{ij}\left(s+t_{n}\right)\left(f_{i}\left(\omega_{i}\left(s+t_{n}\right)\right)-f_{i}\left(\omega_{i}\left(s\right)\right)\right)ds+\\ & & \int_{s_{n}}^{t}\sum_{j=1}^{p}\left(d_{ij}\left(s+t_{n}\right)-d_{ij}\left(s\right)\right)f_{i}\left(\omega_{i}\left(s\right)\right)ds+\int_{s_{n}}^{t}\left(u_{i}\left(s+t_{n}\right)-u_{i}\left(s\right)\right)ds+\int_{s_{n}}^{t}\left(v_{i}\left(s+t_{n}\right)-v_{i}\left(s\right)\right)ds. \end{array}$$

There exist positive numbers $\delta_{1}$ and integers l, k such that, for each i=1,2,⋯,p, the following inequalities are satisfied:

(9) $$\frac{6}{l}<\delta_{1}<\delta ;$$

(10) $$|a_{i}\left(t+s\right)-a_{i}\left(s\right)|<\epsilon_{0}\left(\frac{1}{l}+\frac{2}{k}\right),\;t\in R,$$

(11) $$|c_{ij}\left(t+s\right)-c_{ij}\left(s\right)|<\epsilon_{0}\left(\frac{1}{l}+\frac{2}{k}\right),\;t\in R,$$

(12) $$|u_{i}\left(t+s\right)-u_{i}\left(s\right)|<\epsilon_{0}\left(\frac{1}{l}+\frac{2}{k}\right),\;t\in R,$$

(13) $$\left(m_{i}^{a}+H+m_{i}^{b}+\sum_{j=1}^{p}\left(m_{ij}^{c}+m_{ij}^{d}\right)L+1\right)\left(\frac{1}{l}+\frac{2}{k}\right)<\frac{1}{4},$$

(14) $$|\omega_{i}\left(t+s\right)-\omega_{i}\left(t\right)|<\epsilon_{0}\min\left(\frac{1}{k},\frac{1}{4l}\right),\;t\in R,\left|s\right|<\delta_{1}.$$

Let the numbers $\delta_{1},\;l$ and *k*, as well as numbers n∈N, and i=1,⋯,p, be fixed. Consider the following two alternatives: (i) $|\omega_{i}\left(t_{n}+s_{n}\right)-\omega_{i}\left(s_{n}\right)|\;<\epsilon_{0}/l;$ (ii) $|\omega_{i}\left(t_{n}+s_{n}\right)-\omega_{i}\left(s_{n}\right)|\;\geq \epsilon_{0}/l.$

(i) Using (14), one can show that

(15) $$\begin{array}{ccc} & |\omega_{i}\left(t+t_{n}\right)-\omega_{i}\left(t_{n}\right)| & \leq |\omega_{i}\left(t+t_{n}\right)-\omega_{i}\left(s_{n}+t_{n}\right)|+|\omega_{i}\left(s_{n}+t_{n}\right)-\omega_{i}\left(s_{n}\right)|+|\omega_{i}\left(s_{n}\right)-\omega_{i}\left(t\right)|\\ & & <\frac{\epsilon_{0}}{l}+\frac{\epsilon_{0}}{k}+\frac{\epsilon_{0}}{k}=\epsilon_{0}\left(\frac{1}{l}+\frac{2}{k}\right),\;i=1,2,\cdots ,p, \end{array}$$

if $t\in [s_{n},s_{n}+\delta_{1}].$ Therefore, the condition (C8) and inequalities (9)–(15) imply that

$$\begin{array}{ccc} & & |\omega_{i}\left(t+t_{n}\right)-\omega_{i}\left(t\right)|\geq \int_{s_{n}}^{t}|v_{i}\left(s+t_{n}\right)-v_{i}\left(s\right)|ds-\int_{s_{n}}^{t}|\omega_{i}\left(s\right)\left|\right|b_{i}\left(s+t_{n}\right)-b_{i}\left(s\right)|ds-\\ & & \int_{s_{n}}^{t}\sum_{j=1}^{p}|d_{ij}\left(s+t_{n}\right)-d_{ij}\left(s\right)\left|\right|f_{i}(\omega_{i}\left(s\right)|ds-\int_{s_{n}}^{t}|a_{i}\left(s+t_{n}\right)\left|\right|\omega_{i}\left(s+t_{n}\right)-\omega_{i}\left(s\right)|ds-\\ & & \int_{s_{n}}^{t}|\omega_{i}\left(s\right)||a_{i}\left(s+t_{n}\right)-a_{i}\left(s\right)|ds-\int_{s_{n}}^{t}|b_{i}\left(s+t_{n}\right)\left|\right|\omega_{i}\left(s+t_{n}\right)-\omega_{i}\left(s\right)|ds-\\ & & \int_{s_{n}}^{t}\sum_{j=1}^{p}|c_{ij}\left(s+t_{n}\right)\left|\right|f_{i}\left(\omega_{i}\left(s+t_{n}\right)\right)-f_{i}\left(\omega_{i}\left(s\right)\right)|ds-\int_{s_{n}}^{t}\sum_{j=1}^{p}|c_{ij}\left(s+t_{n}\right)-c_{ij}\left(s\right)\left|\right|f_{i}\left(\omega_{i}\left(s\right)\right)|ds-\\ & & \int_{s_{n}}^{t}\sum_{j=1}^{p}|d_{ij}\left(s+t_{n}\right)\left|\right|f_{i}\left(\omega_{i}\left(s+t_{n}\right)\right)-f_{i}\left(\omega_{i}\left(s\right)\right)|ds-\int_{s_{n}}^{t}|u_{i}\left(s+t_{n}\right)-u_{i}\left(s\right)\left|ds-\right|\omega_{i}\left(s_{n}+t_{n}\right)-\omega_{i}\left(s_{n}\right)|\geq \\ & & \delta_{1}\epsilon_{0}-2\delta_{1}Hm_{i}^{b}-2\delta_{1}\sum_{j=1}^{p}m_{ij}^{d}m_{f}-\delta_{1}m_{i}^{a}\epsilon_{0}\left(\frac{1}{l}+\frac{2}{k}\right)-\delta_{1}H\epsilon_{0}\left(\frac{1}{l}+\frac{2}{k}\right)-\delta_{1}m_{i}^{b}\epsilon_{0}\left(\frac{1}{l}+\frac{2}{k}\right)-\\ & & \delta_{1}\sum_{j=1}^{p}m_{ij}^{c}L\epsilon_{0}\left(\frac{1}{l}+\frac{2}{k}\right)-\delta_{1}\sum_{j=1}^{p}m_{ij}^{d}L\epsilon_{0}\left(\frac{1}{l}+\frac{2}{k}\right)-\delta_{1}\epsilon_{0}\left(\frac{1}{l}+\frac{2}{k}\right)-\frac{\epsilon_{0}}{l}=\\ & & \delta_{1}\left(\epsilon_{0}-2Hm_{i}^{b}-2\sum_{j=1}^{p}m_{ij}^{d}m_{f}-\left(m_{i}^{a}+H+m_{i}^{b}+\sum_{j=1}^{p}\left(m_{ij}^{c}+m_{ij}^{d}\right)L+1\right)\epsilon_{0}\left(\frac{1}{l}+\frac{2}{k}\right)\right)-\frac{\epsilon_{0}}{l}>\frac{\epsilon_{0}}{2l} \end{array}$$

for $t\in [s_{n},s_{n}+\delta_{1}].$

(ii) If $|\omega_{i}\left(t_{n}+s_{n}\right)-\omega_{i}\left(s_{n}\right)|\geq \epsilon_{0}/l$, it is not difficult to find that (14) implies:

(16) $$\begin{array}{ccc} & |\omega_{i}\left(t+t_{n}\right)-\omega_{i}\left(t\right)| & \geq |\omega_{i}\left(t_{n}+s_{n}\right)-\omega_{i}\left(s_{n}\right)|-|\omega_{i}\left(s_{n}\right)-\omega_{i}\left(t\right)|-|\omega_{i}\left(t+t_{n}\right)-\omega_{i}\left(t_{n}+s_{n}\right)|\\ & & >\frac{\epsilon_{0}}{l}-\frac{\epsilon_{0}}{4l}-\frac{\epsilon_{0}}{4l}=\frac{\epsilon_{0}}{2l},\;i=1,2,\cdots ,p, \end{array}$$

if $t\in [s_{n}-\delta_{1},s_{n}+\delta_{1}]$ and n∈N. Thus, it can be concluded that ω(t) is an unpredictable solution with sequences $t_{n},$ $s_{n}$ and positive numbers $\frac{\delta_{1}}{2},$ $\frac{\epsilon_{0}}{2l}.$

Next, we will prove the stability of the solution ω(t). It is true that

$$\begin{array}{c} \omega_{i}\left(t\right)=e^{-\int_{t_{0}}^{t}a_{i}\left(u\right)du}\omega_{i}\left(t_{0}\right)-\int_{t_{0}}^{t}e^{-\int_{s}^{t}a_{i}\left(u\right)du}\left(b_{i}\left(s\right)\omega_{i}\left(s\right)+\sum_{j=1}^{p}\left(c_{ij}\left(s\right)+d_{ij}\left(s\right)\right)f_{j}\left(\omega_{j}\left(s\right)\right)+u_{i}\left(s\right)+v_{i}\left(s\right)\right)ds, \end{array}$$

for all i=1,…,p.

Let $y\left(t\right)=(y_{1}\left(t\right),y_{2}\left(t\right),\cdots ,y_{p}\left(t\right)),$ be another solution of system (1). Then,

$$\begin{array}{c} y_{i}\left(t\right)=e^{-\int_{t_{0}}^{t}a_{i}\left(u\right)du}y_{i}\left(t_{0}\right)-\int_{t_{0}}^{t}e^{-\int_{s}^{t}a_{i}\left(u\right)du}\left(b_{i}\left(s\right)y_{i}\left(s\right)+\sum_{j=1}^{p}\left(c_{ij}\left(s\right)+d_{ij}\left(s\right)\right)f_{j}\left(y_{j}\left(s\right)\right)+u_{i}\left(s\right)+v_{i}\left(s\right)\right)ds, \end{array}$$

for all i=1,…,p.

Making use of the relation:

$$\begin{array}{ccc} & & y_{i}\left(t\right)-\omega_{i}\left(t\right)=e^{-\int_{t_{0}}^{t}a_{i}\left(u\right)du}\left(y_{i}\left(t_{0}\right)-\omega_{i}\left(t_{0}\right)\right)-\int_{t_{0}}^{t}e^{-\int_{t_{0}}^{t}a_{i}\left(u\right)du}(b_{i}\left(s\right)y_{i}\left(s\right)-b_{i}\left(s\right)\omega_{i}\left(s\right)+\\ & & \sum_{j=1}^{p}c_{ij}f_{j}\left(y_{j}\left(s\right)\right)-\sum_{j=1}^{p}c_{ij}f_{j}\left(\omega_{j}\left(s\right)\right)+\sum_{j=1}^{p}d_{ij}f_{j}\left(y_{j}\left(s\right)\right)-\sum_{j=1}^{p}d_{ij}f_{j}\left(\omega_{j}\left(s\right)\right))ds, \end{array}$$

we obtain that:

$$\begin{array}{c} \left|y_{i}\left(t\right)-\omega_{i}\left(t\right)\right|\leq K_{i}e^{-\lambda_{i}\left(t-t_{0}\right)}|y_{i}\left(t_{0}\right)-\omega_{i}\left(t_{0}\right)|+\int_{t_{0}}^{t}K_{i}e^{-\lambda_{i}\left(t-t_{0}\right)}\left(m_{i}^{b}+\sum_{j=1}^{p}\left(m_{ij}^{c}+m_{ij}^{d}\right)L\right)\left|y_{i}\left(s\right)-\omega_{i}\left(s\right)\right|ds, \end{array}$$

for all i=1,2,…,p.

Applying the Gronwall–Belman Lemma, one can obtain:

(17) $$\begin{array}{ccc} & & |y_{i}\left(t\right)-\omega_{i}\left(t\right)\left|\leq K_{i}|y_{i}\left(t_{0}\right)-\omega_{i}\left(t_{0}\right)\right|e^{\left(K_{i}\left(m_{i}^{b}+L\sum_{j=1}^{p}\left(m_{ij}^{c}+m_{ij}^{d}\right)\right)-\lambda_{i}\right)\left(t-t_{0}\right)}, \end{array}$$

for each i=1,2,…,p. So, (C7) implies that $\omega \left(t\right)=(\omega_{1}\left(t\right),\omega_{2}\left(t\right),\cdots ,\omega_{p}\left(t\right))$ is an exponentially stable unpredictable solution of the neural network (1). The theorem is proven. □

## 4. Numerical Examples

Let $\psi_{i},i\in Z,$ be a solution of the logistic discrete equation:

(18) $$\lambda_{i+1}=\mu \lambda_{i}\left(1-\lambda_{i}\right),$$

with μ=3.91.

In the paper [25], an example was constructed of the unpredictable function Θ(t). The function $\Theta \left(t\right)=\int_{-\infty }^{t}e^{-3(t-s)}\Omega \left(s\right)ds,$ where Ω(t) is a piecewise constant function defined on the real axis through the equation $\Omega \left(t\right)=\psi_{i}$ for t∈[i,i+1),i∈Z.

In what follows, we will define the piecewise constant function, Ω(t), for t∈[hi,h(i+1)), where i∈Z and *h* is a positive real number. The number *h* is said to be *the length of step* of the functions Ω(t) and Θ(t). We call the ratio of the period and the length of step, ∇=ω/h *the degree of periodicity*.

Below, using numerical simulations, we will show how the degree of periodicity affects the dynamics of a neural network.

**Example** **1.** *Let us consider the following Hopfield-type neural network:*

(19) $$\begin{array}{c} x_{i}^{\prime }\left(t\right)=-\left(a_{i}\left(t\right)+b_{i}\left(t\right)\right)x_{i}\left(t\right)+\sum_{j=1}^{3}\left(c_{ij}\left(t\right)+d_{ij}\left(t\right)\right)f_{j}\left(x_{j}\left(t\right)\right)+u_{i}\left(t\right)+v_{i}\left(t\right),\; \end{array}$$

**Figure 1:**
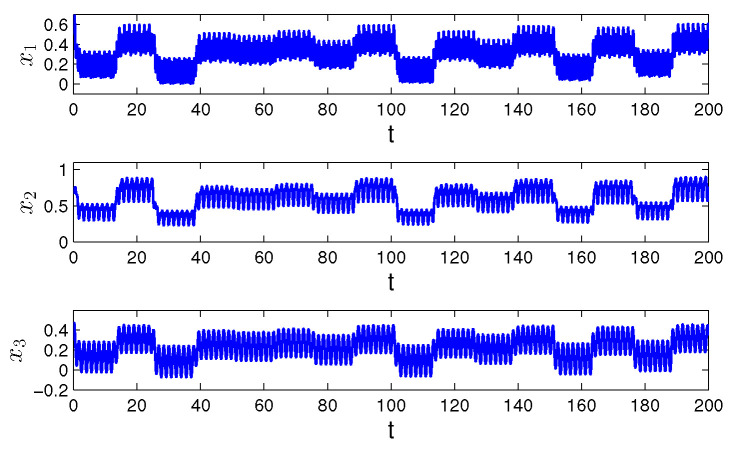
The time series of the coordinates $x_{1}\left(t\right)$, $x_{2}\left(t\right)$ and $x_{3}\left(t\right)$ of the solution of system (19) with the initial conditions $x_{1}\left(0\right)=0.5$, $x_{2}\left(0\right)=0.7$, $x_{3}\left(0\right)=0.3$ and ∇=1/8.

**Figure 2:**
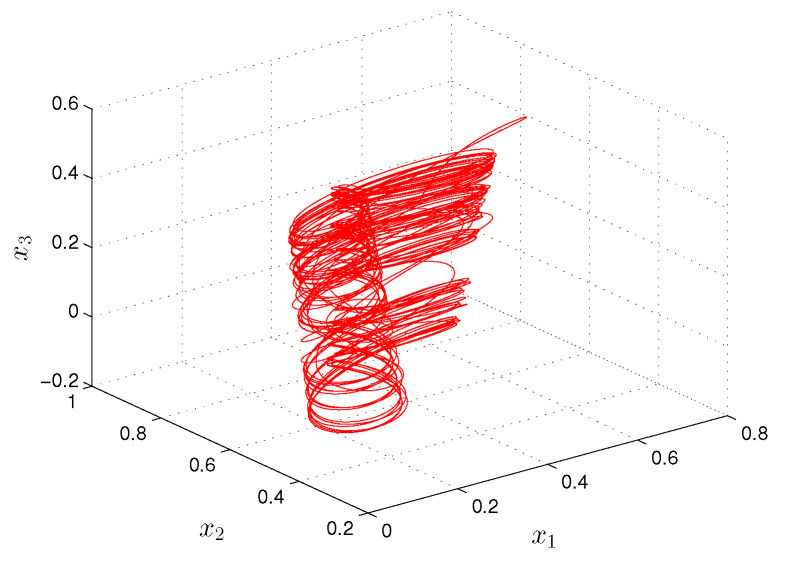
The trajectory of the neural network (19).

 *where i=1,2,3, f(x(t))=0.2tanh(x(t)). The functions $a_{i}\left(t\right),$ $c_{ij}\left(t\right)$ and $u_{i}\left(t\right)$ are π/2—periodic such that $a_{1}\left(t\right)=2+\sin^{2}\left(2t\right),$ $a_{2}\left(t\right)=3+\cos\left(4t\right),$ $a_{3}\left(t\right)=4+\cos^{2}\left(2t\right),$ $c_{11}\left(t\right)=0.1\cos\left(4t\right),$ $c_{12}\left(t\right)=0.3\sin\left(2t\right),$ $c_{13}\left(t\right)=0.1\cos\left(8t\right),$ $c_{21}\left(t\right)=0.2\sin\left(8t\right),$ $c_{22}\left(t\right)=0.05\cos\left(4t\right),$ $c_{23}\left(t\right)=0.4\sin\left(2t\right),$ $c_{31}\left(t\right)=0.3\cos\left(2t\right),$ $c_{32}\left(t\right)=0.5\sin\left(4t\right),$ $c_{33}\left(t\right)=0.1\sin\left(8t\right),$ $u_{1}\left(t\right)=\sin\left(8t\right),$ $u_{2}\left(t\right)=\sin\left(4t\right),$ $u_{3}\left(t\right)=\cos\left(4t\right).$ The unpredictable functions $b_{i}\left(t\right),$ $d_{ij}\left(t\right)$ and $v_{i}\left(t\right)$ such that $b_{1}\left(t\right)=0.2\Theta \left(t\right),$ $b_{2}\left(t\right)=0.6\Theta \left(t\right),$ $b_{3}\left(t\right)=0.4\Theta \left(t\right),$ $d_{11}\left(t\right)=0.02\Theta \left(t\right),$ $d_{12}\left(t\right)=0.05\Theta \left(t\right),$ $d_{13}\left(t\right)=0.03\Theta \left(t\right),$ $d_{21}\left(t\right)=0.04\Theta \left(t\right),$ $d_{22}\left(t\right)=0.01\Theta \left(t\right),$ $d_{23}\left(t\right)=0.06\Theta \left(t\right),$ $d_{31}\left(t\right)=0.06\Theta \left(t\right),$ $d_{32}\left(t\right)=0.06\Theta \left(t\right),$ $d_{33}\left(t\right)=0.05\Theta \left(t\right),$ $v_{1}\left(t\right)=3\Theta \left(t\right),$ $v_{2}\left(t\right)=5\Theta \left(t\right),$ $v_{3}\left(t\right)=4\Theta \left(t\right),$ where $\Theta \left(t\right)=\int_{-\infty }^{t}e^{-2.5(t-s)}\Omega \left(s\right)ds$ with the length of step h=4π. Condition (C1) is valid, and $K_{i}=1,$ i=1,2,3, $\lambda_{1}=5\pi /4,$ $\lambda_{2}=6\pi /4,$ $\lambda_{3}=9\pi /4.$ Since the elements of the convergence sequence are multiples of h=4π, and the period ω is equal to π/2, condition (C3) is valid. The degree of periodicity is equal to 1/8. Conditions (C4)–(C8) are satisfied with H=1, $m_{f}=0.2,$ L=0.2, $m_{1}^{b}=0.08,$ $m_{2}^{b}=0.24,$ $m_{3}^{b}=0.16,$ $m_{11}^{c}=0.1,$ $m_{12}^{c}=0.3,$ $m_{13}^{c}=0.1,$ $m_{21}^{c}=0.2,$ $m_{22}^{c}=0.05,$ $m_{23}^{c}=0.4,$ $m_{31}^{c}=0.3,$ $m_{32}^{c}=0.5,$ $m_{33}^{c}=0.1,$ $m_{11}^{d}=0.008,$ $m_{12}^{d}=0.02,$ $m_{13}^{d}=0.012,$ $m_{21}^{d}=0.016,$ $m_{22}^{d}=0.004,$ $m_{23}^{d}=0.024,$ $m_{31}^{d}=0.024,$ $m_{32}^{d}=0.024,$ $m_{33}^{d}=0.02,$ $m_{1}^{u}=m_{2}^{u}=m_{3}^{u}=1,$ $m_{1}^{v}=1.2,$ $m_{2}^{v}=2,$ $m_{3}^{v}=1.6.$ According Theorem 1, the neural network (19) admits a unique asymptotically stable, unpredictable solution $\omega \left(t\right)=(\omega_{1}\left(t\right),\omega_{2}\left(t\right),\omega_{3}\left(t\right)).$ In Figure 1 and Figure 2, the coordinates and the trajectory of the neural network are shown (19), which asymptotically convergence to the coordinates and trajectory of the unpredictable solution ω(t). Moreover, utilizing (17), we have that:*

$$\begin{array}{c} |x_{1}\left(t\right)-\omega_{1}\left(t\right)|\leq |x_{1}\left(0\right)-\omega_{1}\left(0\right)|e^{-3.62(t-t_{0})}\leq 2e^{-3.62(t-t_{0})},\\ |x_{2}\left(t\right)-\omega_{2}\left(t\right)|\leq |x_{2}\left(0\right)-\omega_{2}\left(0\right)|e^{-4.26(t-t_{0})}\leq 2e^{-4.26(t-t_{0})},\\ |x_{3}\left(t\right)-\omega_{3}\left(t\right)|\leq |x_{3}\left(0\right)-\omega_{3}\left(0\right)|e^{-6.72(t-t_{0})}\leq 2e^{-6.72(t-t_{0})}. \end{array}$$

*Thus, if $t>\frac{1}{3.62}\left(5\ln10+\ln2\right)\approx 3.38,$ then ${∥x\left(t\right)-\omega \left(t\right)∥}_{0}<10^{-5}.$ In other words, what is seen in Figure 1 and Figure 2 for sufficiently large time can be accepted as parts of the graph and trajectory of the unpredictable solution.*

**Example** **2.** *Let us show the simulation results for the following Hopfield-type neural network:*

(20) $$\begin{array}{c} y_{i}^{\prime }\left(t\right)=-\left(a_{i}\left(t\right)+b_{i}\left(t\right)\right)y_{i}\left(t\right)+\sum_{j=1}^{3}\left(c_{ij}\left(t\right)+d_{ij}\left(t\right)\right)f_{j}\left(y_{j}\left(t\right)\right)+u_{i}\left(t\right)+v_{i}\left(t\right),\; \end{array}$$

 *where i=1,2,3, f(y(t))=0.5arctg(y(t)).*

**Figure 3:**
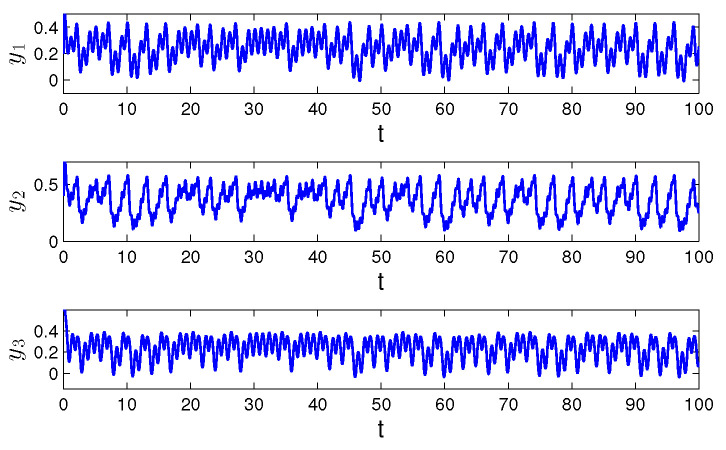
The time series of the coordinates $y_{1}\left(t\right)$, $y_{2}\left(t\right)$ and $y_{3}\left(t\right)$ of the solution of system (20) with the initial conditions $y_{1}\left(0\right)=0.5$, $y_{2}\left(0\right)=0.7$, $y_{3}\left(0\right)=0.3,$ and ∇=1.

**Figure 4:**
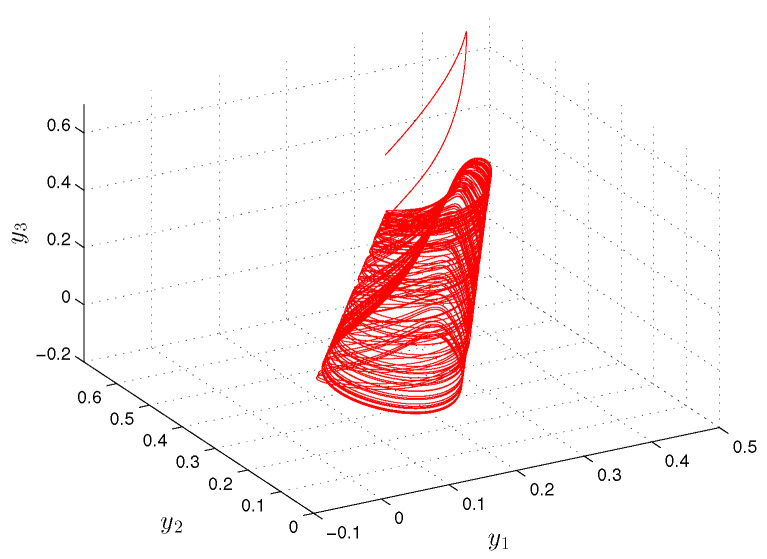
The trajectory of the neural network (20).

*The functions $a_{i}\left(t\right),$ $c_{ij}\left(t\right)$ and $u_{i}\left(t\right)$ are periodic with common period ω=1, and $a_{1}\left(t\right)=5+\cos\left(2\pi t\right),$ $a_{2}\left(t\right)=4+\sin^{2}\left(\pi t\right),$ $a_{3}\left(t\right)=6+0.5\sin\left(2\pi t\right),$ $c_{11}\left(t\right)=0.4\cos\left(2\pi t\right),$ $c_{12}\left(t\right)=0.2\sin\left(4\pi t\right),$ $c_{13}\left(t\right)=0.1\cos\left(8\pi t\right),$ $c_{21}\left(t\right)=0.1\cos\left(4\pi t\right),$ $c_{22}\left(t\right)=0.4\cos\left(2\pi t\right),$ $c_{23}\left(t\right)=0.4\sin\left(4\pi t\right),$ $c_{31}\left(t\right)=0.3\sin\left(2\pi t\right),$ $c_{32}\left(t\right)=0.5\cos\left(4\pi t\right),$ $c_{33}\left(t\right)=0.2\cos\left(2\pi t\right),$ $u_{1}\left(t\right)=\cos\left(2\pi t\right),$ $u_{2}\left(t\right)=0.5\sin\left(4\pi t\right),$ $u_{3}\left(t\right)=\sin\left(2\pi t\right).$ The functions $b_{i}\left(t\right),$ $d_{ij}\left(t\right)$ and $v_{i}\left(t\right)$ are unpredictable such that $b_{1}\left(t\right)=0.5\Theta \left(t\right),$ $b_{2}\left(t\right)=0.3\Theta \left(t\right),$ $b_{3}\left(t\right)=0.7\Theta \left(t\right),$ $d_{11}\left(t\right)=0.3\Theta \left(t\right),$ $d_{12}\left(t\right)=0.6\Theta \left(t\right),$ $d_{13}\left(t\right)=0.2\Theta \left(t\right),$ $d_{21}\left(t\right)=0.3\Theta \left(t\right),$ $d_{22}\left(t\right)=0.5\Theta \left(t\right),$ $d_{23}\left(t\right)=0.3\Theta \left(t\right),$ $d_{31}\left(t\right)=0.1\Theta \left(t\right),$ $d_{32}\left(t\right)=0.2\Theta \left(t\right),$ $d_{33}\left(t\right)=0.5\Theta \left(t\right),$ $v_{1}\left(t\right)=6\Theta \left(t\right),$ $v_{2}\left(t\right)=8\Theta \left(t\right),$ $v_{3}\left(t\right)=7\Theta \left(t\right),$ where $\Theta \left(t\right)=\int_{-\infty }^{t}e^{-3(t-s)}\Omega \left(s\right)ds$ with the length of step h=1. Condition (C1) is valid, and $K_{i}=1,$ i=1,2,3, $\lambda_{1}=5,$ $\lambda_{2}=4.5,$ $\lambda_{3}=6.$ Conditions (C2) and (C3) are satisfied since the elements of the convergence sequence are multiples of h=1 and the period ω is equal to 1. The degree of periodicity equals to 1. Conditions (C4)–(C8) are satisfied with H=1, $m_{f}=\pi /4,$ L=0.5, $m_{1}^{b}=1/6,$ $m_{2}^{b}=1/10,$ $m_{3}^{b}=7/30,$ $m_{11}^{c}=0.4,$ $m_{12}^{c}=0.2,$ $m_{13}^{c}=0.1,$ $m_{21}^{c}=0.1,$ $m_{22}^{c}=0.4,$ $m_{23}^{c}=0.4,$ $m_{31}^{c}=0.3,$ $m_{32}^{c}=0.5,$ $m_{33}^{c}=0.2,$ $m_{11}^{d}=0.1,$ $m_{12}^{d}=0.2,$ $m_{13}^{d}=0.07,$ $m_{21}^{d}=0.1,$ $m_{22}^{d}=0.17,$ $m_{23}^{d}=0.1,$ $m_{31}^{d}=0.34,$ $m_{32}^{d}=0.07,$ $m_{33}^{d}=0.17,$ $m_{1}^{u}=1,$ $m_{2}^{u}=0.5,$ $m_{3}^{u}=1,$ $m_{1}^{v}=2,$ $m_{2}^{v}=8/3,$ $m_{3}^{v}=7/3.$ Figure 3 and Figure 4 demonstrate the coordinates and the trajectory of the solution $y\left(t\right)=(y_{1}\left(t\right),y_{2}\left(t,y_{3}\left(t\right)\right)),$ of the neural network (20), with initial values $y_{1}\left(0\right)=0.2,$ $y_{2}\left(0\right)=0.4,$ $y_{3}\left(0\right)=0.6.$ The solution $y\left(t\right)=(y_{1}\left(t\right),y_{2}\left(t,y_{3}\left(t\right)\right))$ asymptotically converges to the unpredictable solution ω(t). By estimation (17), one can obtain that ${∥y\left(t\right)-\omega \left(t\right)∥}_{0}<10^{-6}$ for $t>\frac{1}{4.175}\left(6\ln10+\ln2\right)\approx 3.48.$*.

**Example** **3.** *Finally, we will show how the degree of periodicity, ∇>1, effects the dynamics of the Hopfield-type neural network:*

(21) $$\begin{array}{c} z_{i}^{\prime }\left(t\right)=-\left(a_{i}\left(t\right)+b_{i}\left(t\right)\right)z_{i}\left(t\right)+\sum_{j=1}^{3}\left(c_{ij}\left(t\right)+d_{ij}\left(t\right)\right)f_{j}\left(z_{j}\left(t\right)\right)+u_{i}\left(t\right)+v_{i}\left(t\right),\; \end{array}$$

**Figure 5:**
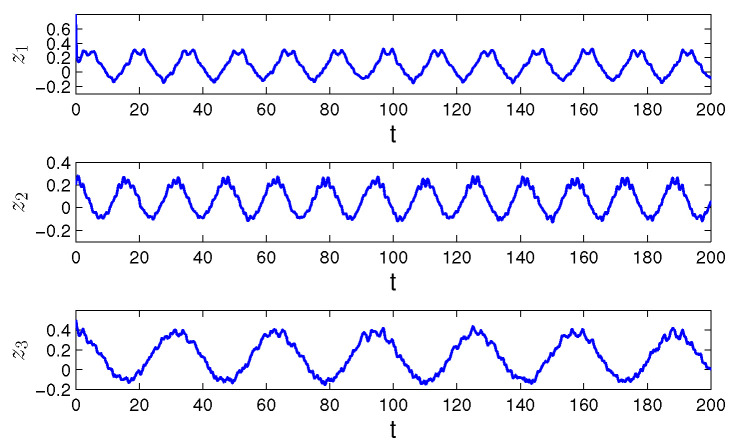
The coordinates $z_{1}\left(t\right)$, $z_{2}\left(t\right)$ and $z_{3}\left(t\right)$ of the solution of system (21) with the initial conditions $z_{1}\left(0\right)=0.8$, $z_{2}\left(0\right)=0.2$, $z_{3}\left(0\right)=0.5$ and ∇=100.

**Figure 6:**
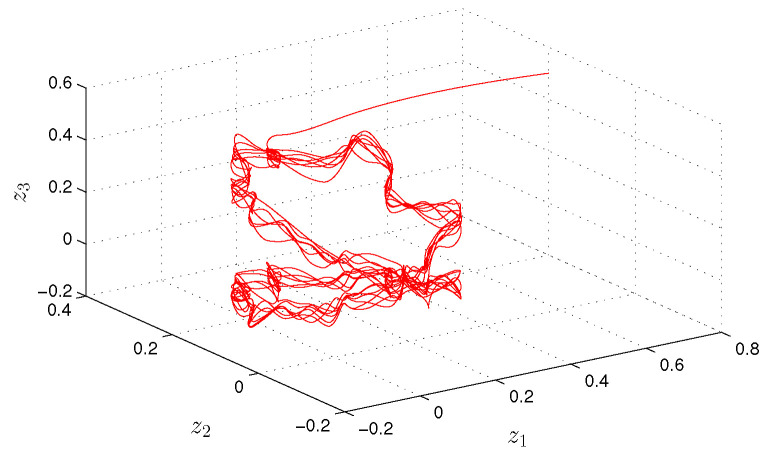
The trajectory of the neural network (21).

 *where i=1,2,3, f(z(t))=0.25arctg(z(t)). The functions $a_{i}\left(t\right),$ $c_{ij}\left(t\right)$ and $u_{i}\left(t\right)$ are periodic with common period ω=10π, and $a_{1}\left(t\right)=5+\sin\left(2t\right),$ $a_{2}\left(t\right)=6+\cos\left(4t\right),$ $a_{3}\left(t\right)=4+0.5\sin\left(2t\right),$ $c_{11}\left(t\right)=0.01\sin\left(2t\right),$ $c_{12}\left(t\right)=0.04\cos\left(4t\right),$ $c_{13}\left(t\right)=0.02\sin\left(8t\right),$ $c_{21}\left(t\right)=0.05\cos\left(4t\right),$ $c_{22}\left(t\right)=0.03\sin\left(2t\right),$ $c_{23}\left(t\right)=0.03\cos\left(8t\right),$ $c_{31}\left(t\right)=0.02\sin\left(4t\right),$ $c_{32}\left(t\right)=0.05\cos\left(2t\right),$ $c_{33}\left(t\right)=0.01\cos\left(4t\right),$ $u_{1}\left(t\right)=\sin\left(0.4t\right),$ $u_{2}\left(t\right)=\cos\left(0.4t\right),$ $u_{3}\left(t\right)=\cos\left(0.2t\right).$ The unpredictable functions $b_{i}\left(t\right),$ $d_{ij}\left(t\right)$ and $v_{i}\left(t\right)$ are such that $b_{1}\left(t\right)=0.8\Theta \left(t\right),$ $b_{2}\left(t\right)=0.3\Theta \left(t\right),$ $b_{3}\left(t\right)=0.4\Theta \left(t\right),$ $d_{11}\left(t\right)=0.04\Theta \left(t\right),$ $d_{12}\left(t\right)=0.05\Theta \left(t\right),$ $d_{13}\left(t\right)=0.02\Theta \left(t\right),$ $d_{21}\left(t\right)=0.05\Theta \left(t\right),$ $d_{22}\left(t\right)=0.01\Theta \left(t\right),$ $d_{23}\left(t\right)=0.06\Theta \left(t\right),$ $d_{31}\left(t\right)=0.01\Theta \left(t\right),$ $d_{32}\left(t\right)=0.06\Theta \left(t\right),$ $d_{33}\left(t\right)=0.03\Theta \left(t\right),$ $v_{1}\left(t\right)=1.6\Theta \left(t\right),$ $v_{2}\left(t\right)=1.4\Theta \left(t\right),$ $v_{3}\left(t\right)=1.8\Theta \left(t\right),$ where $\Theta \left(t\right)=\int_{-\infty }^{t}e^{-2(t-s)}\Omega \left(s\right)ds$ with the length of step h=0.1π. All conditions (C1)–(C8) are valid with $K_{i}=1,$ i=1,2,3, $\lambda_{1}=50\pi ,$ $\lambda_{2}=40\pi ,$ $\lambda_{3}=60\pi ,$ H=1, $m_{f}=\pi /4,$ L=0.25, $m_{1}^{b}=0.4,$ $m_{2}^{b}=0.15,$ $m_{3}^{b}=0.2,$ $m_{11}^{c}=0.01,$ $m_{12}^{c}=0.04,$ $m_{13}^{c}=0.02,$ $m_{21}^{c}=0.05,$ $m_{22}^{c}=0.03,$ $m_{23}^{c}=0.03,$ $m_{31}^{c}=0.02,$ $m_{32}^{c}=0.05,$ $m_{33}^{c}=0.01,$ $m_{11}^{d}=0.02,$ $m_{12}^{d}=0.025,$ $m_{13}^{d}=0.01,$ $m_{21}^{d}=0.025,$ $m_{22}^{d}=0.005,$ $m_{23}^{d}=0.03,$ $m_{31}^{d}=0.005,$ $m_{32}^{d}=0.03,$ $m_{33}^{d}=0.015,$ $m_{1}^{u}=m_{2}^{u}=m_{3}^{u}=1,$ $m_{1}^{v}=0.8,$ $m_{2}^{v}=0.7,$ $m_{3}^{v}=0.9.$ The degree of periodicity is equal to 100. In Figure 5 and Figure 6, we depict the coordinates and the trajectory of the solution $z\left(t\right)=(z_{1}\left(t\right),z_{2}\left(t\right),z_{3}\left(t\right))$ of the neural network (21), with initial values $z_{1}\left(0\right)=0.8,$ $z_{2}\left(0\right)=0.2,$ $z_{3}\left(0\right)=0.5.$ The solution z(t) asymptotically converges to the unpredictable solution ω(t).*

Observing the graphs in Figure 1 and Figure 3, if ∇≤1, we see that the unpredictability prevails. More preciously, periodicity appears only locally on separated intervals if ∇<1, and is not seen at all for ∇=1. Oppositely, if ∇>1, one can see in Figure 5 that the solution admits clear periodic shape, which is enveloped by the unpredictability.

## 5. Conclusions

In this paper, we consider HNNs with variable two-component connection matrix, rates and external inputs. Sufficient conditions are obtained to ensure the existence of exponentially stable unpredictable solutions for HNNs. We introduced and utilized the quantitative characteristic, the degree of periodicity, which differentiates contribution of components, that is, the periodicity and the unpredictability, in the outputs of the model. The obtained results make it possible to find effects of periodicity in chaotic oscillations, which is very important for synchronization, stabilization and control of chaos.

## Data Availability

Not applicable.
